# Linear Laser Scanning Measurement Method Tracking by a Binocular Vision

**DOI:** 10.3390/s22093572

**Published:** 2022-05-07

**Authors:** Chunyan Wu, Li Yang, Zai Luo, Wensong Jiang

**Affiliations:** 1 College of Metrology & Measurement Engineering, China Jiliang University, Hangzhou 310018, China; p20020854086@cjlu.edu.cn (C.W.); jwensong@cjlu.edu.cn (W.J.); 2 College of Information Engineering, China Jiliang University, Hangzhou 310018, China; lyang@cjlu.edu.cn

**Keywords:** stereo vision, 3D metrology, calibration, hybrid measurement, laser

## Abstract

The 3D scanning of a freeform structure relies on the laser probe and the localization system. The localization system, determining the effect of the point cloud reconstruction, will generate positioning errors when the laser probe works in complex paths with a fast speed. To reduce the errors, in this paper, a linear laser scanning measurement method is proposed based on binocular vision calibration. A simple and effective eight-point positioning marker attached to the scanner is proposed to complete the positioning and tracking procedure. Based on this, the method of marked point detection based on image moment and the principle of global coordinate system calibration are introduced in detail. According to the invariance principle of space distance, the corresponding points matching method between different coordinate systems is designed. The experimental results show that the binocular vision system can complete localization under different light intensities and complex environments, and that the repeated translation error of the binocular vision system is less than 0.22 mm, while the rotation error is less than 0.15°. The repeated error of the measurement system is less than 0.36 mm, which can meet the requirements of the 3D shape measurement of the complex workpiece.

## 1. Introduction

In recent years, 3D topography measurement technology has been widely used in cultural relic protection, the aerospace industry, reverse engineering, biomedicine, and other fields [1,2,3,4]. A non-contact measurement—the scanning method based on optics and vision—has been increasingly popular due to its high accuracy, good flexibility, and fast speed [5].

According to the measurement principles, optical-based scanning methods mainly consist of the laser scanning method, the interferometry method, and the structured light method [6,7,8]. The linear laser scanning method is suitable for the measurement of complex hole surfaces based on laser triangulation technology [9]. This method generally requires a mobile platform to complete the scanning measurement. The interferometry method [10] is based on the dual-beam interference, the multi-beam interference, or the holographic interference to generate interference fringes. The geometric shape of the measured object can be measured according to the difference of the fringes. The measurement stability of this method is easily affected by optical vibration, humidity, and other factors. By utilizing image coding technology such as the gray code or the step-by-step phase-shifting method [11], the surface structured light method [12] can restore the 3D coordinate of an object surface to the measurement system. Compared with the linear laser scanning method, this method is more complicated and time-consuming. Therefore, in this paper, the objective is to achieve large-sized 3D measurement with the line laser technique.

Limited by the measurement principle, efficiency, and other issues, however, the field of view of the optical scanning equipment cannot be large enough, which prevents the probe from measuring the topography of a large-sized part directly. Therefore, it is necessary to adopt a hybrid measurement method to expand the measurement range. Also, since the mere local coordinate measurement cannot achieve global unification of measured values, the fusion and conversion methods are required for measured data under different measurement systems [13]. There are three kinds of methods [14] to be applied to the 3D data stitching, and they are as follows: 

The first method is the multi-sensor perspective splicing method. For example, Liu et al. [15] proposed a 3D measurement system to improve the accuracy of point cloud stitching based on the Indoor Global Positioning System (IGPS) [16]. Du et al. [17] proposed a flexible large-scale 3D scanning system assembled by combining a robot, a binocular structured light scanner, and a laser tracker. This method relies on high-accuracy instruments such as the laser tracker and the IGPS to directly measure the pose of the scanning device in the global coordinate system. Apart from its high price, this method has a high measurement accuracy and speed. In addition, the relative position relationship between each sensor needs to be calibrated before use by utilizing this method.

The second method is the mechanical splicing method. For example, Novak et al. [18] proposed a new system for a 3D foot-shape rotating scanning measurement system based on the laser-multiple-line-triangulation principle. Liu et al. [19] introduced the axis-eye calibration algorithm to design a line laser rotating scanning measurement system, aiming to complete the registration of the point cloud in the same coordinate system. The mechanical splicing method mainly uses a moving platform such as the parallel guide rail and the high precision rotating table to complete data splicing with a known moving speed or the angular velocity. The measurement accuracy and the range of this type of method are limited by the motion mechanism.

The last method is the marker-assisted splicing method. For example, Braone et al. [20] developed a stereo vision system to efficiently align 3D point clouds, which allows for an automatic alignment by detecting fiducial markers distributed on overlapping areas of adjacent images. Wang et al. [21] presented a mobile 3D scanning system based on the known marked points. This method relies on the artificial markers to build a connection relationship to complete the conversion from the local measurement coordinate system to the global coordinate system. However, the process of pasting easily affects the surface characteristics of the measured workpiece, which will lead to an error accumulation on the scanning probe.

In addition, the hybrid scanning measurement is improved by scholars for different application areas and work scenarios. For example, Chen et al. [22] proposed a two-stage binocular vision system to measure the relative pose between two components, aiming to reduce coordinate transformation errors. Yin et al. [23] proposed a free-moving surface reconstruction technique based on the binocular structured light. This method can eliminate the movement constraints of the parallel guide rail and reduce the accumulation of error but is not suitable for large-sized objects. Liu et al. [24] built a binocular structured light system combined with a wide field of view camera, and a plane target was used as an intermediary to realize the alignment of local point cloud data. Shi et al. [25] proposed a 3D scanning measurement method based on a stereoscopic tracker. The position alignment of the scanner in different views was calculated by tracking the LED markers fixed to the scanner. However, in the case of occlusion, LED markers may be seriously deformed, which will result in inaccurate positioning. Hu et al. [26] proposed a new real-time catadioptric stereo tracking method, which can realize stereo measurement under monocular vision. Huang et al. [27] designed a new 3D scanner with a zoom lens unit, which realized large-area scanning at a low magnification rate and high-precision detail scanning at a high magnification rate. The two scanning results complement each other to ensure that the system has both high reconstruction accuracy and a large scanning area. Jiang et al. [28] proposed a system calibration method to reduce measurement errors caused by scale differences, which is suitable for combined measurement systems with different scales and ranges.

In order to enhance the flexibility of hybrid measurement, a method for localized surface scanning measurement is proposed by using circular markers to position the laser scanner. The main contribution lies in the design of a new circular mark recognition method and a coordinate transformation calibration method in the combined system. The proposed circle detection method based on image moments takes the weighted mean of the centroid of multiple circles as observation points, which can reduce the center deviation error and improve positioning accuracy. Different from the existing work, the laser scanner can be accurately located without fully identifying all the marked points, which can overcome the limitation of local occlusion and enhance the robustness of the system to a certain extent. Finally, through the calibration of each coordinate system, the alignment of 3D point cloud data at different angles is completed.

The remainder of this paper is structured as follows. Section 2 introduces the structure of the measurement system. Section 3 introduces the measurement principle and detection algorithms in the positioning process. In Section 4, the proposed method is verified through calibration experiments and measurement experiments, and concluding remarks are provided in Section 5.

## 2. Overview of the Laser Scanning System

**Figure 1:**
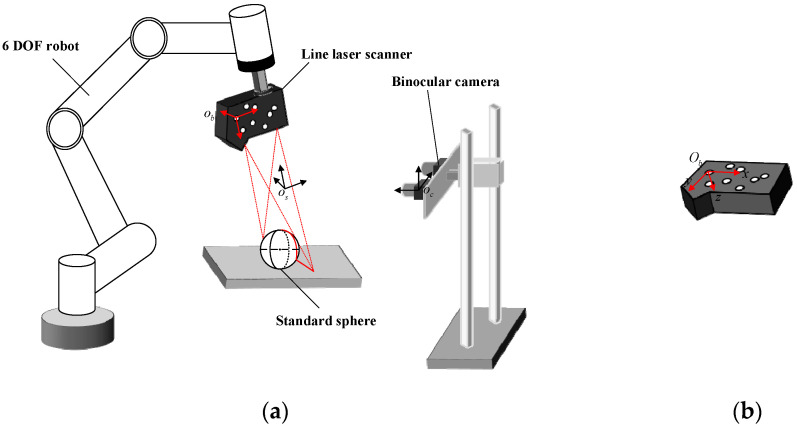
(**a**) System composition and structure diagram. (**b**) The markers and the marker coordinate system.

The laser scanning system is designed based on binocular vision and mainly consists of a laser scanner, a 6 DOF robot, and a binocular camera. The structure and composition of the system are shown in Figure 1a. The laser scanner is fixed on the end of the robotic arm through a flange. As a significant mark for the binocular camera, the circular marked points are randomly pasted on the surface of the laser scanner to determine the pose of the laser scanner. The binocular camera is installed at a suitable distance to ensure that the circular marked points can be recognized.

In order to facilitate the positioning of the scanner, eight circular marked points are randomly pasted on the scanner to achieve robust and precise positioning. The inner diameters of the eight circular marking points are 10 mm. With the first center point in the upper left corner as the starting point, each circle is numbered in a clockwise direction. In addition, the first center point of the upper left corner is used as the reference point to establish the marker coordinate system, as shown in Figure 1b. The x-axis is parallel to the upper surface of the scanner, and the y-axis is parallel to the right side of the scanner. The three axes are perpendicular to each other, which satisfies the right-hand rule.

**Figure 2:**
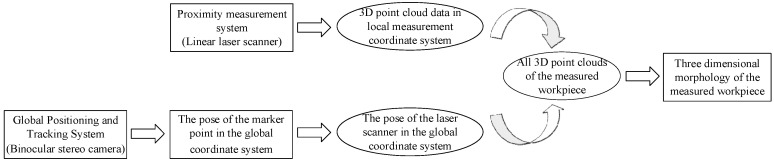
Flow chart of linear laser scanning measurement.

In the process of scanning, the laser scanner moves in any direction under the drive of the mechanical arm to complete the scanning measurement of the workpiece. The point cloud data, based on the scanner measurement coordinate system, is constantly changing with the scanning process. In order to complete the splicing of all point cloud data, it is necessary to transform point cloud data to a fixed coordinate system. The process of unifying point cloud data to the same coordinate system is shown in Figure 2.

The scanner measurement coordinate system is assumed as the laser coordinate system (LCS), which is the local coordinate system, noted as $O_{s}-X_{s}Y_{s}Z_{s}$. The marker coordinate system (MCS) is expressed by $O_{b}-X_{b}Y_{b}Z_{b}$. The camera coordinate system (CCS) is expressed by $O_{c}-X_{c}Y_{c}Z_{c}$, which is the global coordinate system. The point cloud data in the local coordinate system is mainly unified to the global coordinate system through the following equation:

(1) $$Pc=\left[\begin{array}{cc} R_{b}^{c} & t_{b}^{c}\\ 0 & 1 \end{array}\right]\left[\begin{array}{cc} R_{s}^{b} & t_{s}^{b}\\ 0 & 1 \end{array}\right]Ps$$

where Ps is the 3D coordinate of the surface point of the workpiece in LCS, Pc is the 3D coordinate of the corresponding point in CCS. $R_{s}^{b}$ and $t_{s}^{b}$ are denoted as the rotation matrix and the translation vector from LCS to MCS, respectively, and $R_{b}^{c}$ and $t_{b}^{c}$ are assumed as the rotation matrix and the translation vector from MCS to CCS, respectively.

## 3. Measurement Principle and Related Algorithms

### 3.1. Marker Detection

The laser scanner of our suggested measurement system is fixed at the end of the robot through a flange. However, the laser scanner has fewer textures, and the features on it are not obvious. In order to facilitate the positioning of the scanner, eight circular marked points are randomly pasted on the scanner to achieve robust and precise positioning. As a significant mark for calculating the pose of the scanner, these marked points are rigidly connected to the scanner. The accuracy of the pose calculation depends on the detection of the marked points.

**Figure 3:**
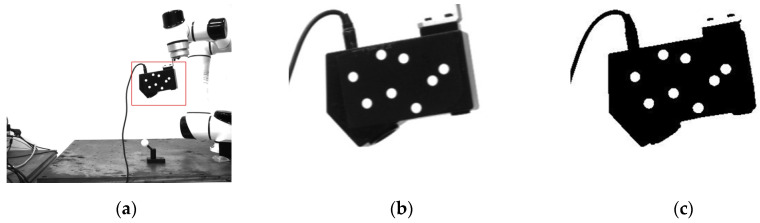

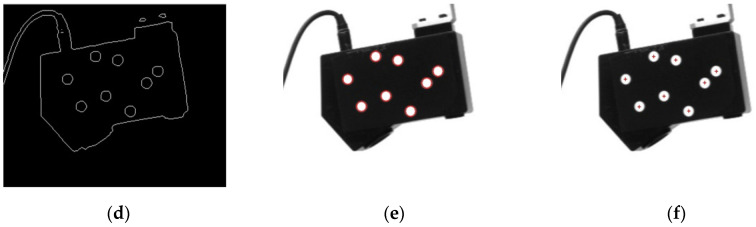
(**a**) Template matching image. (**b**) Region of interest image. (**c**) Binary image. (**d**) Edge detection image. (**e**) Contours image. (**f**) Center marked image.

In this paper, the principle of image moments is applied to complete the center detection of the marked points. The specific methods are as follows:

1. For the original image after correction, the normalized template matching algorithm is used to find the region of interest containing eight marked points. The region of interest is marked with a red rectangular box in the original image, as shown in Figure 3a. The pixel resolution of the ROI is 308×247, and the position of the ROI changes, but its size remains unchanged in the process of movement.
2. The region of interest (Figure 3b) is processed by binarization threshold to obtain the binarization image (Figure 3c). According to the edge contour detection algorithm [29], the edge contours are extracted, as shown in Figure 3d. Each closed connected region is regarded as a separate contour region. Due to the affine transformation of the image, the standard circle is shaped like an ellipse in the image. The contour of the circular marker points can be obtained by filtering the geometric characteristics such as the area and the circularity, as shown in Figure 3e.
3. For each individual contour region, the centroid position of each contour is determined by the image moment property as the initial centroid result.
  (2) $$\bar{x}=\frac{\sum_{i=1}^{M}\sum_{j=1}^{N}\left(x_{i}\times f(x_{i},y_{j})\right)}{\sum_{i=1}^{M}\sum_{j=1}^{N}\left(f(x_{i},y_{j})\right)},\bar{y}=\frac{\sum_{i=1}^{M}\sum_{j=1}^{N}\left(y_{j}\times f(x_{i},y_{j})\right)}{\sum_{i=1}^{M}\sum_{j=1}^{N}\left(f(x_{i},y_{j})\right)}$$
  where $f(x_{i},y_{j})$ is the gray value of the pixel in the *i*-th row and *j*-th column.
4. Since the method of calculating the center by the image moment property is greatly affected by gray scale, to enhance the stability and reliability of the results, the binarization threshold interval of the image is set as [80,200] according to experience, and the binarization threshold is successively accumulated with 10 steps to process the image. Step 2 and step 3 are repeated K times, and the contour of the marked points for each image after threshold processing is detected. According to the inertia ratio, the confidence score of each detection is set, and the final center position of the marker is determined by weighted summation.
  (3) $$\gamma =\frac{(mu20+mu02)-\sqrt{{\left(mu20-mu02\right)}^{2}+{\left(2mu11\right)}^{2}}}{(mu20+mu02)+\sqrt{{\left(mu20-mu02\right)}^{2}+{\left(2mu11\right)}^{2}}}$$
  where γ is the inertial ratio of the contour, $mu20=\sum_{i=1}^{M}\sum_{j=1}^{N}\left({\left(x_{i}-\bar{x}\right)}^{2}\times f(x_{i},y_{j})\right)$, $mu02=\sum_{i=1}^{M}\sum_{j=1}^{N}\left({\left(y_{j}-\bar{y}\right)}^{2}\times f(x_{i},y_{j})\right)$, $mu11=\sum_{i=1}^{M}\sum_{j=1}^{N}\left((x_{i}-\bar{x})\times (y_{j}-\bar{y})\times f(x_{i},y_{j})\right)$.

For each circular contour, multiple center point detection results can be obtained according to the threshold detection. $\alpha =\gamma^{2}$ is introduced to represent the confidence score of detection, and the final center point position is expressed as

(4) $$P(\bar{x},\bar{y})=\frac{\sum_{i=1}^{K}\left(P({\bar{x}}_{i},{\bar{y}}_{i})\times \alpha_{i}\right)}{\sum_{i=1}^{K}\alpha_{i}}$$

Therefore, the central position of each marked point on the image can be determined, as shown in Figure 3f.

### 3.2. Binocular Parallel Stereo Vision Model

When an object is measured by the monocular camera model [30], its mapping relationship between the imaging plane coordinate system and the world coordinate system can be written as

(5) $$z_{c}\left[\begin{array}{c} u\\ v\\ 1 \end{array}\right]=K_{3\times 3}{\left[R,t\right]}_{3\times 4}\left[\begin{array}{c} x_{w}\\ y_{w}\\ z_{w}\\ 1 \end{array}\right]=\left[\begin{array}{ccc} f_{\alpha } & 0 & cx\\ 0 & f_{\beta } & cy\\ 0 & 0 & 1 \end{array}\right]{\left[R,t\right]}_{3\times 4}\left[\begin{array}{c} x_{w}\\ y_{w}\\ z_{w}\\ 1 \end{array}\right]$$

where $K_{3\times 3}$ is the internal parameter of the camera, $f_{\alpha }=f/dx,\;f_{\beta }=f/dy$, f is the focal length of the camera. *dx* and *dy* are the physical size of each pixel in the *x* and *y* directions, respectively. (cx,cy) is the optical center coordinate of the camera, ${\left[R,t\right]}_{3\times 4}$ is the homogeneous transformation matrix from the world coordinate system to the camera coordinate system, (u,v) is the pixel coordinates on the imaging plane, $\left(x_{w},y_{w},z_{w}\right)$ is the 3D coordinates of the corresponding point in world space, and $z_{c}$ is the depth factor.

**Figure 4:**
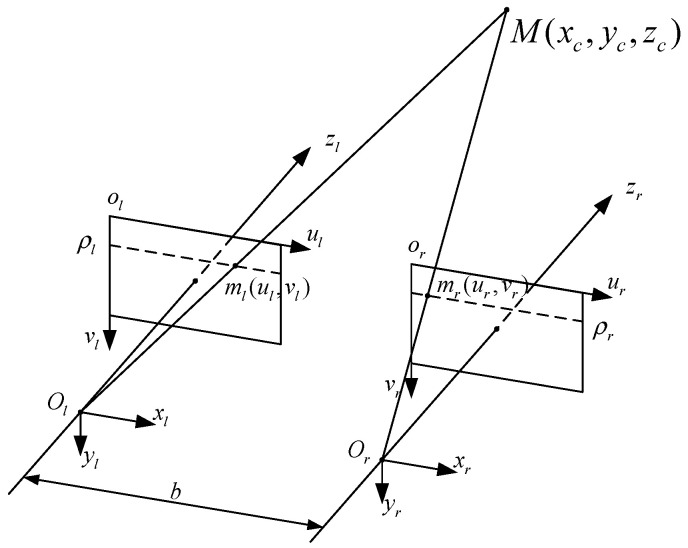
Schematic diagram of binocular parallel stereo vision.

In the binocular parallel stereo vision model, the optical axes of the left camera and the right camera are parallel and perpendicular to the baseline, satisfying the epipolar constraint, as shown in Figure 4. The optical center distance of two cameras is defined as the baseline, noted as b. $M(x_{c},y_{c},z_{c})$ is the 3D coordinate of the feature point in the camera coordinate system, $\left(u_{l},v_{l}\right)$ is the coordinate in the left camera image coordinate system, and $\left(u_{r},v_{r}\right)$ is the coordinate in the right camera image coordinate system. According to the principle of the similar triangle, the 3D coordinates in the new camera coordinate system can be obtained by using Equation (6) as

(6) $$\{\begin{array}{c} x_{c}=b\frac{u_{l}}{u_{l}-u_{r}}\\ y_{c}=b\frac{v}{u_{l}-u_{r}}\\ z_{c}=b\frac{f}{u_{l}-u_{r}} \end{array}$$

In Equation (6), $v=v_{l}=v_{r}$ is the y-pixel coordinate of the feature point.

The internal parameters of the camera are obtained by the camera calibration, which transforms the object from the 3D coordinates on the world coordinate system to the image plane. The 3D coordinates are unified in the newly corrected camera coordinate system based on the binocular stereo vision model. From this, the 3D coordinates of the marked points in CCS can be obtained.

### 3.3. Homonymy Point Matching Algorithm

For the images containing the laser scanner captured by the cameras, the center of the circle is extracted as a feature point through the detection method in Section 3.1. The epipolar constraint is exploited to correlate the corresponding feature points in the left and right images. Finally, through the parallax model in Section 3.2, the 3D coordinates of the circular marked points in CCS can be obtained.

Completing homonymy point matching of MCS and CCS is a key step in calculating the position conversion of these two coordinate systems. According to the principle of space distance invariance, a method for homonymy point matching is designed. By utilizing this matching method, the location of the laser sensor can be efficiently and quickly determined at each moment.

There are four steps for the matching algorithm, and they are as follows:

1. Building the reference distance library. According to the measurement calibration system, *n* three-dimensional coordinates of the marked points based on MCS can be calculated. These coordinates form a coordinate set as follows:
  (7) $$\Omega_{pw}=\{P_{1},P_{2},\cdots ,P_{n}\}\;\;P_{i}=(x_{iw},y_{iw},z_{iw}),i=1,2,\cdots ,n$$

**Figure 5:**
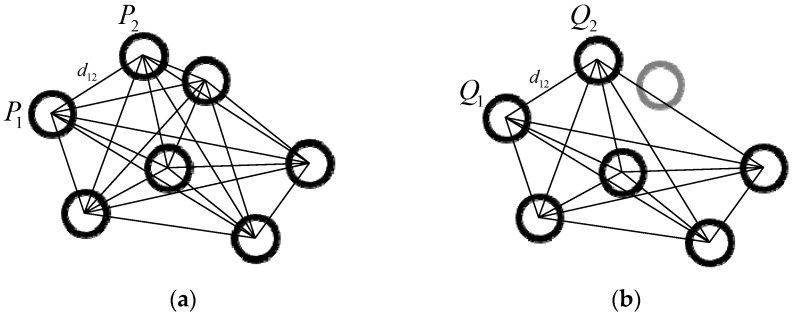
(**a**) Schematic diagram of the reference distance library. (**b**) Schematic diagram of the sample distance library.

The Euclidean distance relationship between these points is represented, as shown in Figure 5a. The distance between every two marked points is calculated, and the reference distance library are composed, as follows:

(8) $$\Omega_{WD}=\{WD_{1},WD_{2},\cdots ,WD_{n}\}\;\;WD_{i}=\{d_{ij}\},j=1,2,\cdots ,n$$

where $WD_{i}$ is the set of the distances from the *i*-th point to other points and $d_{ij}$ is the distance between the *i*-th point and the *j*-th point. The elements in each subset WD are arranged in ascending order.

2. Building the sample distance library. After image processing on the images with marked points captured by the left camera and the right camera, m(m≤n) 3D coordinate sets of marked points in CCS are obtained as follows:
  (9) $$\Omega_{pc}=\{Q_{1},Q_{2},\cdots ,Q_{m}\}\;\;Q_{t}=(x_{tc},y_{tc},z_{tc}),t=1,2,\cdots ,m$$

The Euclidean distance representation between sample points is shown in Figure 5b. The distance between every two marked points is calculated, and the sample distance library is composed as follows:

(10) $$\Omega_{CD}=\{CD_{1},CD_{2},\cdots ,CD_{m}\}\;\;CD_{t}=\{d_{tk}\},k=1,2,\cdots ,m$$

Similarly, the elements in each subset CD are also arranged in ascending order.

3. Matching the corresponding points. According to the principle of invariance of spatial distance, the matching method of points with the same name is realized. The Euclidean distance between $Q_{1}$ and $Q_{2}$ is expressed by δ. Considering the influence of the error that the distance between the points reconstructed by the camera may deviate from the true distance, ε is assumed as this distance error threshold. The elements satisfying the range d∈[δ−ε,δ+ε] in $\Omega_{WD}$ are found and denoted as $\left\{d_{ab}\right\}$, where a and b are the integers from 1 to n. Then, the possible initial matching point set of $Q_{1}$ is $\left\{P_{a},P_{b}\right\}$. In order to further determine the corresponding point of $Q_{1}$ in MCS, the number of subsets in $WD_{*}$ that satisfy Equation (11) is counted and denoted as x. If x is greater than m−1, it means that the corresponding point of $Q_{1}$ in MCS is $P_{*}$. In the same way, the corresponding points of the remaining m−1 marked points in MCS can be determined.
  (11) $$\left|CD_{1}(t)-WD_{*}(i)|\leq \varepsilon \;\;,*\in \{a,b\right\}$$
4. Calculating the conversion relationship. According to the previous steps, the 3D coordinates of the corresponding marked points in the two different coordinate systems of MCS and CCS can be obtained; they are expressed by $P_{c}=\{p_{c1},\cdots ,p_{cm}\}$ and $P_{b}=\{p_{b1},\cdots ,p_{bm}\}$, respectively. The problem is transformed into a point cloud registration problem with known matching information, which is to find an optimal Euclidean transformation to minimize the error.
  (12) $$\underset{R,t}{\min}\frac{1}{2}\sum_{i=1}^{m}{\left\|\left(p_{ci}-(Rp_{bi}+t)\right)\right\|}_{2}^{2}$$

There are many closed-form solutions for this type of optimization, and the principles of each solution are basically similar. Here, the SVD decomposition algorithm in the literature [31] is used to calculate the pose parameters.

### 3.4. Hand–Eye Calibration of LCS and MCS

In the process of scanning, the laser scanner and the marked points move together without changing their relative positions. Therefore, the conversion of LCS and MCS does not change, which can be regarded as a rigid body transformation. The determination of the relationship between LCS and MCS is similar to the hand–eye calibration model [32]. It can be obtained by measuring the same point by changing the position of the line laser scanner multiple times. However, one disadvantage of the laser scanner is that it cannot directly and accurately obtain the coordinates of specific feature points. Combined with its measurement characteristics, a calibration model with the center of the standard sphere as the feature point is established. In addition to obtaining the transformation relationship, another parameter result of this calibration method is the coordinate value of the center of the standard sphere in the global coordinate system, which can be used to verify the quality of the calibration result.

**Figure 6:**
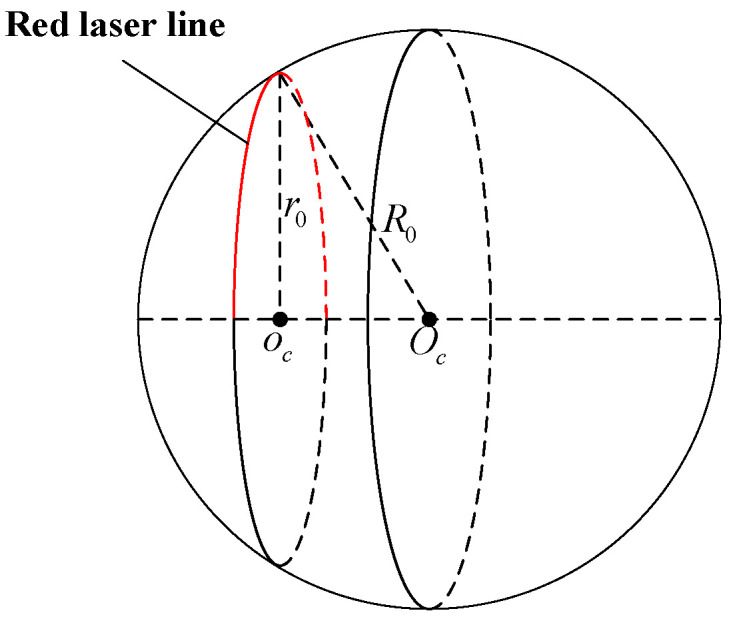
Schematic diagram of standard sphere calibration.

When the laser scanner tests the standard sphere, the laser is attached to the spherical surface to obtain point cloud data, as shown in Figure 6. The value on the x-axis of these point cloud data is 0. According to the point cloud data, the center coordinate $o_{c}=(0,y_{0},z_{0})$ and the radius $r_{0}$ under this arc can be fitted. Combining the spatial geometric relationship between the circle center coordinates $o_{0}$, the arc radius $r_{0}$, and the sphere radius $R_{0}$, the 3D coordinates of the center of the sphere in LCS can be calculated as $P_{s}(x_{s},y_{s},z_{s})$.

(13) $$\{\begin{array}{c} x_{s}=\pm \sqrt{R_{0}^{2}-r_{0}^{2}}\\ y_{s}=y_{0}\\ z_{s}=z_{0} \end{array}$$

For the flexible 3D measurement system, $P_{c}(x_{c},y_{c},z_{c})$ is denoted as the 3D coordinate of the center of the standard sphere in CCS and $P_{s}(x_{s},y_{s},z_{s})$ is assumed as the 3D coordinate of the corresponding point in LCS. The local 3D coordinates measured by the laser scanner can be integrated into the camera coordinate system using Equation (14):

(14) $$P_{c}=R_{b}^{c}(R_{s}^{b}P_{s}+t_{s}^{b})+t_{b}^{c}$$

where $R_{s}^{b}$ and $t_{s}^{b}$ are the rotation matrix and the translation vector from LCS to MCS, respectively. Similarly, $R_{b}^{c}$ and $t_{b}^{c}$ are the rotation matrix and the translation vector from MCS to CCS.

**Figure 7:**
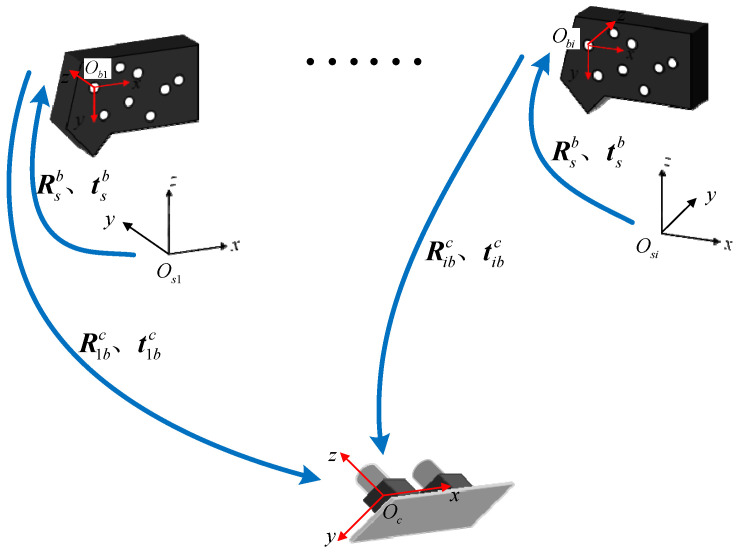
Coordinate systems during calibration.

During the calibration process, without changing the position of the standard sphere and binocular vision positioning system, the coordinate of the center of the standard sphere in CCS remains unchanged. However, with the help of the robot to drive the laser scanner to move to different positions, the coordinate of the center of the sphere in LCS is changed. Therefore, as shown in Figure 7, when the scanner is moved to a different position, in combination with Equation (14), multiple sets of conversion equations can be obtained as follows:

(15) $$P_{c}=R_{ib}^{c}(R_{s}^{b}P_{is}+t_{s}^{b})+t_{ib}^{c}$$

where $R_{ib}^{c}$ and $t_{ib}^{c}$ are the rotation matrix and the translation matrix from MCS to CCS in the *i*-th measurement, $P_{is}$ is the coordinate of the center of the standard sphere based on LCS in the *i*-th measurement.

Let $R_{s}^{b}=[r_{1},r_{2},r_{3}]$, where $r_{1},r_{2},r_{3}$ are each column vector of the rotation matrix. Defining that $R_{v}=vec(R_{s}^{b})={\left[r_{1},r_{2},r_{3}\right]}^{T}$, $R_{v}$ is a vector with nine rows and one column. Then, the above equation can be expressed as

(16) $$(P_{is}⊗R_{ib}^{c})\times R_{v}+R_{ib}^{c}t_{s}^{b}-P_{c}=-t_{ib}^{c}$$

where the symbol ⊗ is defined as the Krone product of two matrices, and $P_{is}⊗R_{ib}^{c}$ is a matrix with three rows and nine columns.

Taking $R_{v}$, $t_{s}^{b}$, and $P_{c}$ as the variables to be solved, Equation (16) can be transformed into

(17) $$AX=b\;A_{3n\times 15}=\left(\begin{array}{ccc} P_{1s}⊗R_{1b}^{c} & R_{1b}^{c} & -I_{3\times 3}\\ \vdots & \vdots & \vdots \\ P_{ns}⊗R_{nb}^{c} & R_{nb}^{c} & -I_{3\times 3} \end{array}\right),X_{15\times 1}=\left(\begin{array}{c} R_{v}\\ t_{s}^{b}\\ P_{c} \end{array}\right),b_{3n\times 1}=\left(\begin{array}{c} -t_{1b}^{c}\\ \vdots \\ -t_{nb}^{c} \end{array}\right)$$

where n is the number of measurements in the calibration process, $I_{3\times 3}$ is an identity matrix with three rows and three columns.

For Equation (17), the result can be obtained by using the least squares method as

(18) $$X={\left(A^{T}A\right)}^{-1}A^{T}b$$

Finally, considering the rotation matrix and the translation vector from LCS to MCS, the 3D coordinate of the standard sphere in CCS can be obtained through this calibration model.

### 3.5. Overall Unification of Point Cloud Data

The point cloud data in LCS is mainly unified to CCS through the following equation.

(19) $$Pc=\left[\begin{array}{cc} R_{b}^{c} & t_{b}^{c}\\ 0 & 1 \end{array}\right]\left[\begin{array}{cc} R_{s}^{b} & t_{s}^{b}\\ 0 & 1 \end{array}\right]Ps$$

where $R_{s}^{b}$ and $t_{s}^{b}$ are obtained through the hand–eye calibration model in Section 3.4, which represents the conversion relationship from LCS to MCS. $R_{b}^{c}$ and $t_{b}^{c}$ are calculated by the matching method in Section 3.3, which represent the conversion relationship from MCS to CCS. As a result, driven by the robot, the laser scanner scans the measured workpiece in all directions to complete the 3D topography measurement of the object.

## 4. Experiment and Analysis 

**Figure 8:**
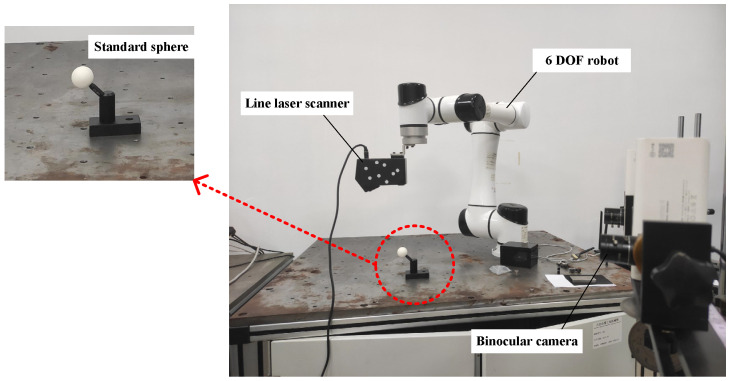
Physical diagram of system configuration.

The physical diagram of the whole system construction is shown in Figure 8. The binocular vision system consists of two industrial cameras. The model of the industrial cameras is JHSM130Bs, with a frame rate of 30 fps and a resolution of 1280 × 1024 pixels. The focal length of the industrial fixed focus lens is 4mm. The linear laser scanner is an nxSensor-I with a frame rate of 15 fps. A maximum of 960 points can be obtained in one scan measurement.

Regarding the laser scanner used in this paper, its internal placement structure is such that the optical axis of the laser is perpendicular to the surface of the measured object, and the optical axis of the camera is at an angle of 55° with the laser plane. This geometry is suitable for measuring objects with small surface height differences and can obtain a higher resolution [33]. For the laser measurement system used in this paper, the single measurement distance is not more than 200 mm, the scanning depth distance in the z-axis direction is 225–345 mm, the resolution in the depth direction can reach 0.0050 mm, the accuracy is 0.045 mm, and the minimum movement displacement of the driving system is 0.050 mm.

### 4.1. Accuracy Experiment for the Binocular Reconstruction

The checkerboard with 9 × 6 corner points is applied to calibrate the internal and external parameters of the two cameras. The side length of each small square of the checkerboard is 30 mm. The internal and external parameters obtained by the calibration are as shown in Table 1. It takes about two minutes to calibrate the binocular camera with a checkerboard.

The position conversion relationship from the right camera to the left camera is as follows. The unit of translation vector in the pose relationship is the millimeter.

$$R_{lr}=\left[\begin{array}{ccc} 0.999503 & -0.022021 & -0.022570\\ 0.021810 & 0.999717 & -0.009537\\ 0.022773 & 0.009040 & 0.999670 \end{array}\right],\;t_{lr}=\left[\begin{array}{c} -264.449\\ -7.38219\\ -2.20070 \end{array}\right]$$

**Figure 9:**
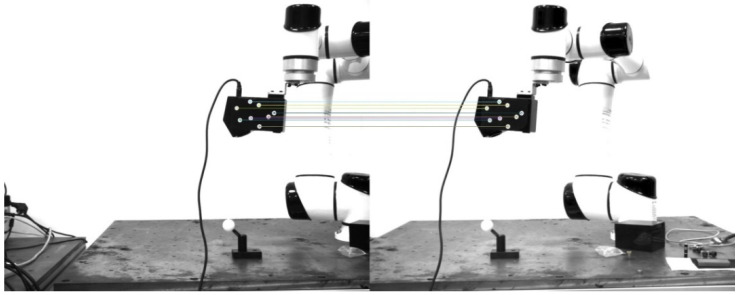
The matching result of the left image and the right image at a certain moment.

After calibration, the reprojection error of the camera is 0.14 pixel, and the epipolar error is 0.18 pixel. According to the calibrated camera parameters, the eight marked points randomly pasted on the surface of the laser scanner can be accurately positioned. The order of the marked points is as follows. Taking the first marked point in the upper left corner as the starting point, then the remaining marked points are sequentially numbered in a clockwise direction. In the positioning experiment, the matching result of the binocular camera at a certain moment is shown in Figure 9. The left and right pixel coordinates of the marked points at a certain moment are shown in Table 2.

For the eight marked points, the TRITOP optical measurement system developed by the German Company GOM has high measurement accuracy and can directly measure the center coordinates of the circular marked points. The TRITOP system consists of one DSLR camera with the largest resolution of 4288 × 2824 pixels, a fixed focal length of 35 mm, contrast coded and uncoded points, and scale bars. In the measurement process, the scale bars are placed on both sides of the object to be measured, and the contrast coded points are pasted on the surface of the object to be measured. The digital camera is used to take continuous photos from different perspectives, and then the 3D coordinates of the circular marked points can be obtained by processing these photos with its own software. Considering the high measurement accuracy of the TRITOP optical measurement system, the system is used to measure the 3D coordinates of these eight marked points, and the Euclidean distance between the two marked points is calculated as the reference value. The binocular positioning system built in this paper is used to perform 71 random positioning experiments on the laser scanner. The results of the first 12 groups are shown in Table 3.

**Figure 10:**
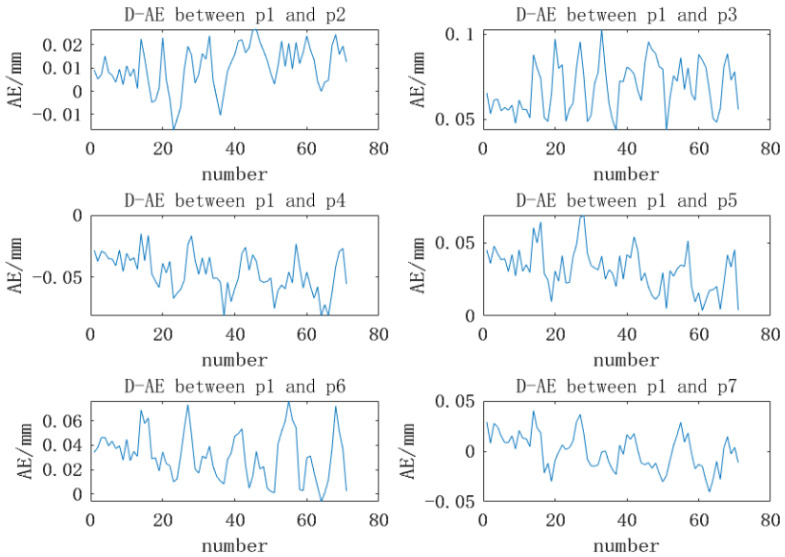
Comparison of the reconstruction distance and the actual distance.

In these 71 experiments, the comparison curve between the positioning distance of the marked points and the actual distance is shown in Figure 10.

The AVG denotes the average of the measured values, the RMSE denotes the root mean square error, the AE denotes the absolute error, the D-AE denotes the absolute error of the distance between two points, and the pi denotes the *i*-th marked point. It can be seen from Table 4 and Figure 10 that the reconstruction measurement of the binocular positioning system has good repeatability and reliability, and the reconstruction accuracy is high. The absolute error is less than 0.08 mm, and the root mean square error is better than 0.01 mm, which can ensure the high-precision positioning of the marked points. It is the basis for the accuracy of the subsequent calculation of the position.

### 4.2. Evaluation of Binocular Positioning Performance

For the laser scanning measurement system based on binocular positioning proposed in this paper, ensuring the accuracy of positioning in different environments is the premise to complete high-precision 3D reconstruction. Therefore, this part mainly evaluates the positioning accuracy of the positioning system from the lightness, distance, viewing angle, shelter, and other factors. Here, with the aid of a 6-DOF mechanical arm as the experimental mobile platform, the rotation accuracy of the arm is 0.003°, and the translation accuracy is 0.03 mm.

#### 4.2.1. Localization Evaluation under Light Changes

**Figure 11:**
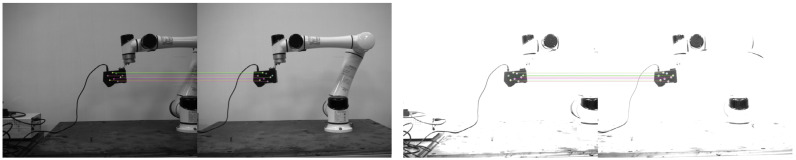
Positioning images with exposure times of 400 µs and 3200 µs.

In this part, the influence of different lightness on the positioning performance of eight-point markers is analyzed. Different lighting conditions are simulated mainly by changing the exposure time of the camera. The binocular camera is placed 1.6 m away from the laser scanner, the initial exposure time research range is set as 400–3200 µs, and the step interval is set as 100 µs. 29 groups of data are collected in the whole experiment, with 30 pairs of images in each group. The average pose solved by these 30 images is taken as the pose solution value under the current exposure time. Among them, the pose is expressed in the form of a Euler Angle, which is divided into rotations and translations in x, y, and z directions, represented by rx, ry, rz, tx, ty, and tz, respectively. Figure 11 shows the image recorded at a partial exposure time. Especially in the first pair of images in Figure 11, in a relatively dark environment, the eight markers may not be fully recognized, but the pose can still be calculated via the correct matching of some points.

**Figure 12:**
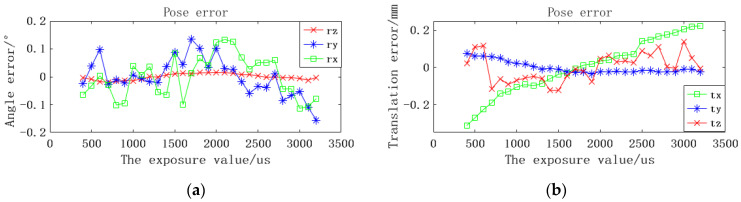
Positioning errors under different brightness conditions. (**a**) Rotational errors on the x, y, and z axes. (**b**) Translation errors on the x, y, and z axes.

Because the positions of the binocular camera and scanner have not moved, the pose obtained from the theoretical analysis should be relatively consistent. The average value of 29 groups of data within the exposure range is taken as the reference value to calculate the deviation between the solved pose and the reference value under each exposure condition. The experimental results are shown in Figure 12. 

As can be seen in Figure 12, even under the influence of different exposure factors, the interference of solving the pose is small. The solution of the angle pose in the z direction is the most stable, with an error of less than 0.015°. The translation deviation in the y and z directions is also less affected, with an error of less than 0.076 mm and 0.12 mm, respectively. The maximum angle deviation in the x direction is 0.11°, and the maximum angle deviation in the y direction is 0.16°, both of which appear under high exposure conditions. However, the lightness condition has a great influence on the pose calculation in the x direction, and the error increases with the increase of brightness. The reason for this may be that when the brightness increases, the extracted circular contour may become smaller, leading to the deviation of the center of the circle.

#### 4.2.2. Positioning Accuracy at Different Distances

**Figure 13:**
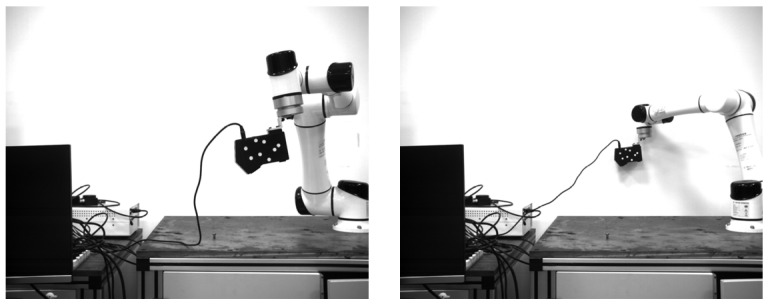
Left camera images at distances of 1.2 m and 2.3 m.

In this section, the positioning accuracy of the system at different distances is mainly studied. Referring to the experiment in the previous section, in order to have better recognition results, the exposure time of the camera is set as 1200 µs in subsequent experiments. The research range of positioning detection distance is set as 1.2–2.3 m. With the industrial mechanical arm as the moving platform, 30 frames of pictures are recorded every 0.1 m of movement along the optical axis of the camera, and the average value is taken as the current value. Figure 13 shows some experimental images. The first frame of each set of data is used as the initial pose, and then the relative poses of the other images are obtained. The Euclidean distance of the translation error is used as a comparison, and the calculation formula is as follows:

(20) $$\Delta t=\sqrt{t_{x}\times t_{x}+t_{y}\times t_{y}+t_{z}\times t_{z}}$$

**Figure 14:**
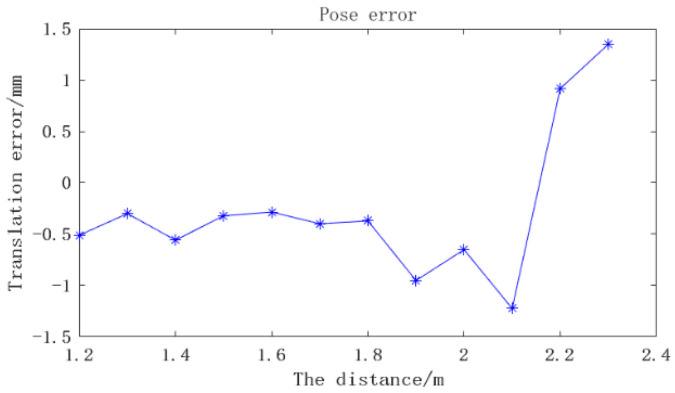
Translation errors at different distances.

As can be seen in Figure 14, the translation deviation of the positioning system is relatively stable within the range of 1.8 m, and the error is less than 0.50 mm. When the distance increases to 2.3 m, the translation error reaches 1.34 mm. The possible reason for this is that at a long distance, the size of the circular mark is too small to be easily identified, resulting in a large error.

#### 4.2.3. Positioning Accuracy at Different Angles

**Figure 15:**
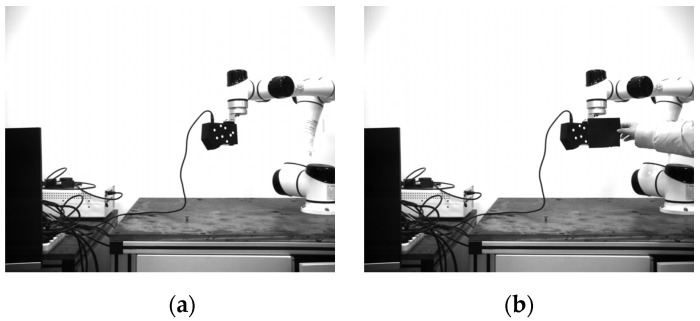
Left camera image at a rotation angle of 30°. (**a**) In the case of no occlusion. (**b**) In the case of the occlusion of two marker points.

**Figure 16:**
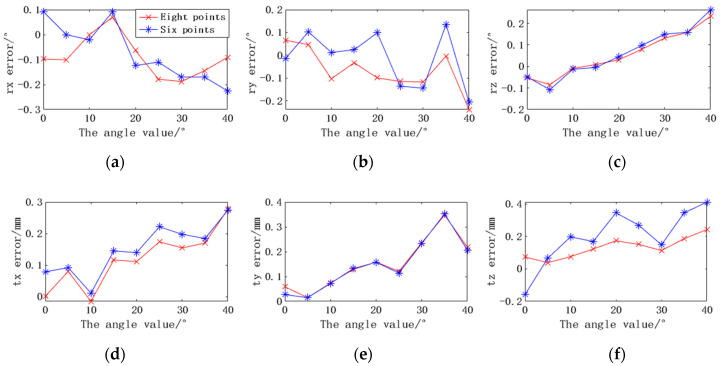
The pose errors based on eight markers and six markers at different angles. (**a**) Rotation error along the x-axis. (**b**) Rotation error along the y-axis. (**c**) Rotation error along the z-axis. (**d**) Translation error along the x-axis. (**e**) Translation error along the y-axis. (**f**) Translation error along the z-axis.

The localization accuracy of the localization system under different angles and occlusions is demonstrated in detail in this section. The binocular camera is placed 1.6 m away from the laser scanner. With the y axis of the camera as the rotation axis, the scanner rotates from 0 to 40°. Ten pairs of continuous images are recorded at each rotation of 5°. In order to compare the robustness of anti-occlusion, two circular markers are artificially blocked at the same position, and ten images are also recorded. Figure 15 shows some experimental images at a rotation angle of 30°. The first frame of each set of data is used as the initial pose, and then the relative poses of the other images are obtained. Then, the average errors of each degree of freedom are calculated. The experimental results are shown in Figure 16.

In Figure 16, when there is no occlusion, the camera can fully recognize eight points. The maximum rotation errors in the x, y, and z directions are −0.19°, −0.24°, and 0.23°, respectively. The maximum translation errors are 0.28 mm, 0.35 mm, and 0.25 mm, respectively. In the case of artificial occlusion, the camera can only locate the scanner through six markers. The maximum average errors of rx, ry, and rz are −0.23°, −0.21°, and 0.26°, and the maximum average errors of tx, ty, and tz are 0.27 mm, 0.35 mm, and 0.41 mm, respectively. The experimental results show that the positioning accuracy does decrease when the marker is partially occluded. The most influential is the translation error in the z-direction, which has increased from 0.25 mm to 0.41 mm. The rotation error has little effect, and the rotation error in both cases is basically kept below 0.26°.

### 4.3. Hand–Eye Calibration Experiment

(1) The standard sphere with a diameter of 30.0055 mm is placed in a suitable position. The binocular camera is installed at a suitable location to ensure that the laser scanner is within the measurement range of the field of view of the positioning system.
(2) The robot is controlled to drive the linear laser scanner to move to a certain position, where the laser beam can be effectively projected on the surface of the standard sphere. Afterward, the line point cloud data can be obtained, and the images taken by the left camera and the right camera are obtained at the same time.
(3) Step (2) is repeated N times (N > 8).
(4) The line point cloud data on the surface of the standard sphere is extracted for fitting, and then the coordinate of the center of the sphere can be calculated in the current laser coordinate system. According to the matching algorithm in Section 3.3, the circular marked points are recognized, and the spatial position changes of the marked points are calculated.
(5) According to the standard sphere calibration model established in Section 3.4, the conversion relationship between LCS and MCS is calculated as follows:
  $$R_{s}^{b}=\left[\begin{array}{ccc} 0.996745 & 0.00427368 & 0.00807998\\ 0.0146129 & -0.00129409 & -0.989261\\ 0.0293743 & 0.999739 & 0.0376670 \end{array}\right],\;t_{s}^{b}=\left[\begin{array}{c} 89.5174\\ 270.536\\ 14.8889 \end{array}\right]$$

At the same time, the 3D coordinate of the center of the standard sphere in CCS during the calibration process is as follows:

$$P_{c}={\left[35.9700,216.343,1303.39\right]}^{T}$$

The whole calibration process takes about 10 min. During the process, it takes 9 min to obtain the point cloud data of multiple groups of different poses by moving the scanner, and 189 ms for the transformation matrix obtained via program processing.

**Figure 17:**
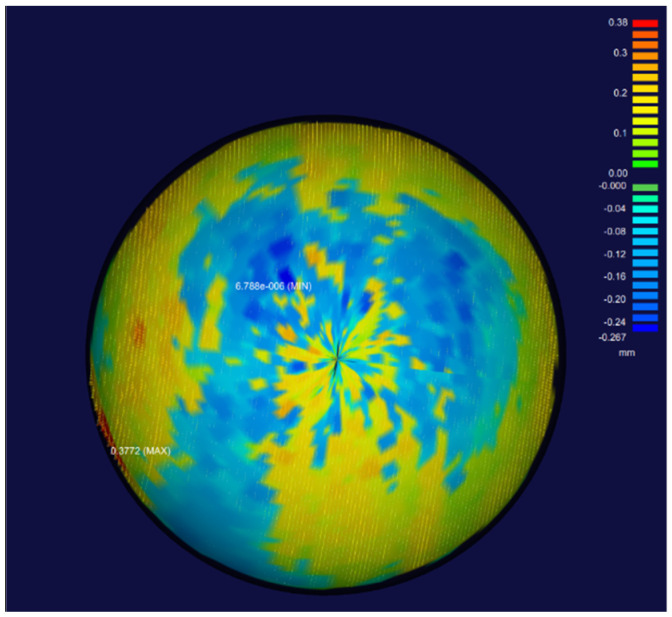
Deviation analysis between the standard sphere model and the scanned point cloud.

In order to verify the calibration accuracy of the system, without changing the position of the standard sphere, the standard sphere is scanned and measured using the calibrated position relationship. Combined with the known diameter of the standard sphere and the sphere center coordinate obtained by the calibration, the standard sphere model is established. The deviation analysis between the scanning point cloud and the sphere fitted by the standard sphere is shown in Figure 17.

In Figure 17, the parts from green to red represent positive deviation, and the parts from green to blue represent negative deviation. The result shows that the error of the point cloud near the edge of the standard sphere is relatively large, the maximum positive deviation is 0.38 mm, and the maximum negative deviation is −0.27 mm. The possible reason for this is that the data obtained at the edge of the scanner is relatively unstable due to the limitation of the scanner principle. The geometric standard deviation is 0.043 mm, showing that the overall deviation is more evenly distributed.

### 4.4. System Spatial Measurement Accuracy Experiment

**Figure 18:**
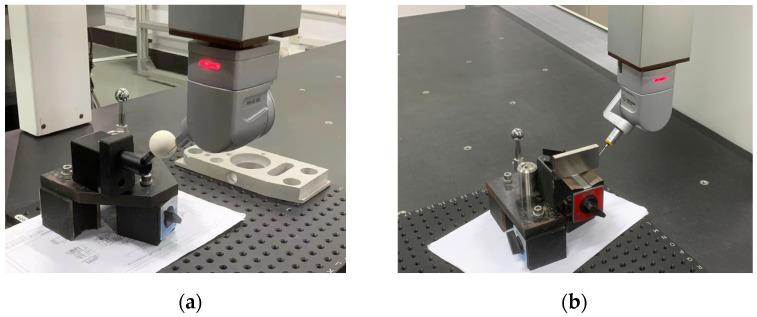
Images of CMM measuring standard parts. (**a**) Measuring standard sphere. (**b**) Measuring standard gauge block.

In order to determine the spatial measurement accuracy of the system, several measurement experiments are carried out with the standard parts. The standard parts used mainly include a standard sphere with a diameter of 30.0055 mm and a standard gauge block with a length of 70 mm. The accuracy of standard parts is 0.0015 mm. To verify the reliability of the standard part data, ten measurement experiments were carried out on the standard ball and standard gauge block using a three-coordinate measuring machine (CMM). The model of the bridge-type CMM is Global 09.15.08 from Hexagon, and the indication error is 1.2 µm. Figure 18 shows photos of the process of using CMM to measure the standard part, and Table 5 records the data of the diameter of the standard ball and the length of the standard gauge block measured by CMM.

It can be determined from the data in Table 5 that the values measured by the CMM are relatively stable and reliable. The average diameter of the measurement standard sphere is 30.002 mm, and the average radius value of the standard sphere is 15.001 mm. The average length of the measurement standard block is 70.000 mm. The value of the standard part is calibrated, and the two average values are taken as the reference true value of the standard part, which is used to compare with the measurement data of the system.

**Figure 19:**
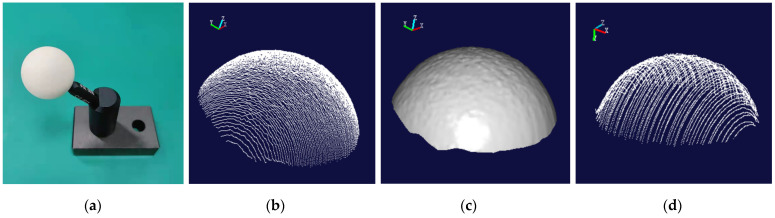
The related images of the standard sphere. (**a**) The original image. (**b**) The point cloud image based on binocular camera positioning. (**c**) The rendering image based on (**b**). (**d**) Point cloud image based on robot positioning.

**Figure 20:**
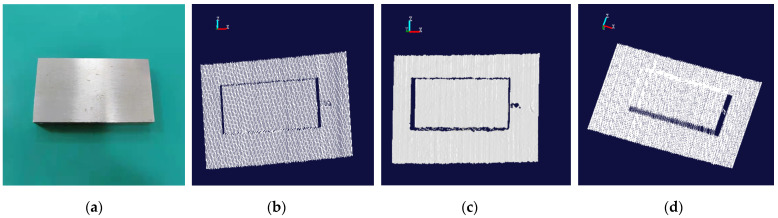
The related images of the standard gauge block. (**a**) The original image. (**b**) The point cloud image based on binocular camera positioning. (**c**) The rendering image based on (**b**). (**d**) Point cloud image based on robot positioning.

According to the calibrated position relationship, the position of the standard sphere and the standard gauge block is fixed, and the scanner measures the gauge block and the standard sphere in a variety of different postures. At the same time, the positioning of the 6-DOF robot itself is used to scan and measure the measuring block and the standard sphere for comparison [34]. The point cloud image and the rendering image of the standard sphere are shown in Figure 19. Similarly, the point cloud image and the rendering image of the standard gauge block are shown in Figure 20. 

According to the laser point cloud data obtained at different positions each time, the radius of the fitted sphere is obtained. The scanner is moved to different positions to measure the radius of the standard sphere and the length of the standard gauge block. Twelve measurement results are recorded in Table 6. The data measured by these two methods are compared with the true value data measured by the CMM, and the standard deviation and root mean square error of the system measurement are calculated. The data are shown in Table 7.

The SD is the standard deviation. It can be seen from Table 7 that within the measurement range of 70 mm, the standard deviation is less than 0.17 mm, indicating that the measurement system has good stability. Compared with the splicing results using robot positioning, our average measurement error is smaller. Compared with the true value of the CMM, the root mean square error of the measurement system does not exceed 0.20 mm, the maximum measurement error for the standard sphere does not exceed 0.19 mm, and the maximum measurement error for the standard gauge block does not exceed 0.36 mm. In summary, referring to the maximum measurement deviation, the spatial measurement accuracy of the system can be determined to be 0.36 mm.

### 4.5. Scanning Measurement Application Experiment

**Figure 21:**
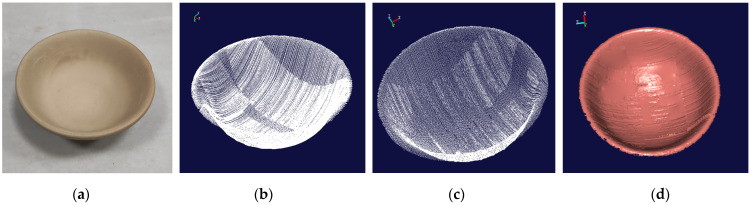
The related images of the ceramic bowl. (**a**) the original image. (**b**) the original point cloud image. (**c**) the point cloud image after down-sampling. (**d**) the rendering image.

In order to verify the feasibility of the entire scanning measurement system, this system is applied to scan and reconstruct the ceramic bowl. The robot is used to drive the laser scanner to scan the ceramic bowl according to the planned route. In the entire scanning process, the linear laser scans 1194 times, and 977,292 points are obtained. The point cloud image of the scanned ceramic bowl is obtained as shown in Figure 21b. Due to the path setting, repeated measurements are made in some parts of the ceramic bowl, resulting in dense point clouds in some parts. With the distance tolerance of 0.5 mm as the constraint, the original point cloud image is downsampled to obtain the downsampled image as shown in Figure 21c. The downsampled image is rendered by “Imageware” software, and the 3D curved surface shape of the ceramic bowl is obtained as shown in Figure 21d.

It can be seen in Figure 21b,c that there are fewer point clouds at the side of the bowl with larger curvatures, indicating that more instances of measurement are required to obtain complete point cloud data at the curved location. In the repeated measurement area, the overlap degree of point cloud is relatively high, showing that the measurement system has good repeatability. In Figure 21d, the scanned point cloud data can fully recover the 3D shape of the measured object, and the overall stitching effect is good, indicating that the measurement system has the 3D measurement capability of a continuous surface.

## 5. Conclusions

This paper mainly introduces a laser scanning measurement system based on binocular vision positioning and its calibration method. Compared to traditional methods, the circular marker is designed to avoid sticking marked points on the measured workpiece. The complex calibration process is not required, and the positioning accuracy is not limited by the motion mechanism. The proposed circle detection method based on image moments takes the weighted mean of the centroid of multiple circles as observation points, which can reduce the center deviation error and improve positioning accuracy. This method is also suitable for the rapid detection and recognition of other circular markers. Compared to a specific target, the marker we designed overcomes the limitation that it cannot be located due to local occlusion, and the laser scanner can be accurately located without completely identifying all the marked points. The measurement range of the positioning system is wider. Therefore, the system can be conveniently applied to measure the 3D shape of large-sized workpieces on site with complex features.

Regarding the eight-point markers designed in this paper: (1) A corresponding marked points detection method is designed based on the image moment properties, which enhances the accuracy of detection. (2) A matching method of spatial points is built by both the epipolar constraint and the principle of the invariance of spatial distance. (3) The mathematical relationship between the laser point cloud and the global coordinates is obtained by a standard spherical calibration model. The marker coordinate system is used as an intermediate bridge to complete the data unification. The system is used to measure the ceramic bowl to verify the practicability and reliability of the measurement system. The experimental results show that the binocular vision system can complete localization under different light intensities and complex environments, the repeated translation error of the binocular vision system is less than 0.22 mm, and the rotation error is less than 0.15°. The repetition error of the measurement system is less than 0.36 mm, and the average measurement result is better than the splicing method relying on the self positioning of the manipulator, which can complete the precise positioning of the scanner and meet the requirements of 3D shape measurement.

The eight-point marking method in the paper also has some limitations. When the measurement distance is large, the marker is too small for positioning, and the size of the circular marker can be increased in the follow-up study. When the rotation angle of the scanner is too large and the number of marked points identified by the positioning system is less than five, it is easy to cause inaccurate positioning. In the follow-up work, we try to solve this problem by sticking circular markers on multiple surfaces of the scanner, so that the binocular positioning system can still recognize more circular markers when the scanner rotates at a large angle, and also the flexibility of the system can be further enhanced. 

## Figures and Tables

**Table 1:** Internal parameter coefficients of two cameras.

	$\left(f_{x},f_{y}\right)$	(cx,cy)	$k_{1}$	$k_{2}$
Left camera	(1621.110, 1621.330)	(653.257, 516.830)	−0.042	0.286
Right camera	(1636.350, 1634.640)	(652.691, 504.998)	0.017	0.055

**Table 2:** The coordinate value of the marked points at a certain time.

The Number of Marked Points	Left Camera Pixel Coordinates	Right Camera Pixel Coordinates	Space Coordinates in CCS
1	(697.376, 442.240)	(356.923, 442.371)	(29.000, −53.269, 1365.262)
2	(762.921, 433.678)	(422.075, 433.779)	(79.841, −59.864, 1363.679)
3	(737.057, 423.396)	(396.589, 423.487)	(59.832, −67.924, 1365.186)
4	(807.995, 456.506)	(466.361, 456.598)	(114.562, −42.052, 1360.543)
5	(791.867, 467.259)	(450.198, 467.327)	(102.062, −33.731, 1360.402)
6	(765.867, 495.424)	(423.979, 495.553)	(81.877, −11.891, 1359.516)
7	(739.902, 471.748)	(398.578, 471.839)	(61.887, −30.276, 1361.771)
8	(707.569, 477.463)	(366.483, 477.565)	(36.851, −25.861, 1362.724)

**Table 3:** Measurement results of the distance between the marked points.

No.	$d_{12}/mm$	$d_{13}/mm$	$d_{14}/mm$	$d_{15}/mm$	$d_{16}/mm$	$d_{17}/mm$	$d_{18}/mm$
1	34.139	51.292	86.424	75.786	67.388	40.280	28.624
2	34.136	51.280	86.415	75.777	67.392	40.258	28.624
3	34.137	51.288	86.423	75.789	67.400	40.278	28.631
4	34.145	51.289	86.422	75.784	67.400	40.275	28.631
5	34.138	51.282	86.417	75.780	67.394	40.266	28.625
6	34.137	51.284	86.417	75.781	67.397	40.259	28.626
7	34.134	51.282	86.412	75.772	67.391	40.259	28.621
8	34.134	51.285	86.424	75.783	67.394	40.266	28.632
9	34.133	51.275	86.407	75.769	67.382	40.253	28.616
10	34.141	51.288	86.422	75.787	67.399	40.271	28.631
11	34.136	51.283	86.416	75.772	67.381	40.264	28.623
12	34.140	51.283	86.418	75.776	67.389	40.263	28.625

**Table 4:** Comparison of the distance measured result with the actual value.

	$d_{12}/mm$	$d_{13}/mm$	$d_{14}/mm$	$d_{15}/mm$	$d_{16}/mm$	$d_{17}/mm$	$d_{18}/mm$
AVG	34.140	51.296	86.407	75.773	67.386	40.251	28.620
Real	34.130	51.227	86.453	75.742	67.354	40.251	28.714
RMSE	0.010	0.015	0.016	0.015	0.020	0.018	0.017

**Table 5:** Measurement results measured with CMM.

No.	1	2	3	4	5	6	7	8	9	10
Diameter/mm	30.004	30.003	30.001	30.001	30.003	30.006	29.997	29.999	30.003	30.005
Length/mm	70.000	69.997	70.001	70.000	70.000	69.998	69.998	70.001	70.000	70.000

**Table 6:** Measurement results of the standard sphere radius and gauge block length.

No.	Radius *R*/mm		Length *L*/mm	
	Our System	Robot Positioning	Our System	Robot Positioning
1	14.958	15.236	70.241	70.170
2	15.125	15.157	69.883	70.236
3	15.004	15.100	70.358	70.215
4	15.182	15.118	70.215	69.876
5	15.091	15.152	70.163	70.129
6	14.963	15.085	69.943	70.174
7	15.166	14.983	70.136	69.954
8	15.029	15.034	70.082	70.190
9	14.916	15.082	69.755	70.143
10	14.900	15.041	69.926	70.091
11	15.079	14.994	70.202	69.984
12	15.050	15.066	70.156	70.230

**Table 7:** Comparison of the measured value with the actual value.

	Radius *R*/mm		Length *L*/mm	
	Our System	Robot Positioning	Our System	Robot Positioning
Average	15.039	15.088	70.088	70.116
Real	15.001	15.001	70.000	70.000
RMSE	0.097	0.111	0.190	0.162
SD	0.090	0.069	0.168	0.112

## Data Availability

The data used to support the findings of this study are available from the first author upon request.
